# Strain-Dependent High-Temperature Low-Cycle Fatigue Behavior and Damage Mechanisms of GH4169 Alloy After Cold-Drawing Pre-Deformation and Short-Time Annealing

**DOI:** 10.3390/ma19214649

**Published:** 2026-10-31

**Authors:** Haiquan Liu, Xinyu Gu, Senhao Yang, Ting Xiong, Yanzhong Tian, Meng Wang, Feng Liu

**Affiliations:** 1 Testing Center, Ji Hua Laboratory, Foshan 528200, China; liuhaiquan@jihualab.ac.cn (H.L.); 15937482943@163.com (S.Y.); 2 Guangdong Provincial Key Laboratory of Advanced Corrosion-Resistant Materials, Institute of Corrosion Science and Technology, Corrosion-Resistant Centre for Light Metal, Guangzhou 510530, China; xygu@icost.ac.cn; 3 Institute of Advanced Materials and Technology, Guangdong University of Technology, Guangzhou 510006, China; xiongting0813@163.com; 4 Key Laboratory for Anisotropy and Texture of Materials (Ministry of Education), School of Materials Science and Engineering, Northeastern University, Shenyang 110819, China; tianyanzhong@mail.neu.edu.cn

**Keywords:** GH4169 alloy, cold-drawing pre-deformation, short-time annealing, low-cycle fatigue, strain-amplitude dependence

## Abstract

The high-temperature low-cycle fatigue (LCF) response of GH4169 (Inconel 718) is sensitive to the initial microstructural state, yet the coupled effects of cold-drawing pre-deformation and subsequent annealing remain insufficiently understood. A direct-aged condition was used as the reference, while specimens subjected to 14% and 20% cold drawing were tested either without annealing (Z) or after short-time annealing at 930 °C for 10 min (T). Strain-controlled LCF tests were conducted at 650 °C at total strain amplitudes of 0.4%, 0.6%, and 0.8%, with EBSD, SEM, and TEM used to characterize microstructures and fatigue damage. The highest recorded N_f_ values were 13,679 cycles for 20%-Z at 0.4%, 1095 cycles for the direct-aged reference at 0.6%, and 289 cycles for 20%-T at 0.8%. Retained deformation substructures and low-angle grain boundaries may contribute to local slip accommodation at low strain amplitude. The dislocation structures observed after testing at 0.4% and 0.6% suggest more extensive localized cyclic plasticity at the higher amplitude, while partial recovery after annealing may benefit high-amplitude plastic accommodation. These results suggest that the balance between retained deformation substructures and partial recovery contributes to the strain-amplitude-dependent LCF response of GH4169.

## 1. Introduction

GH4169 alloy (Inconel 718) is widely used in critical hot-section aero-engine components, including turbine disks and casings, because of its high-temperature strength, microstructural stability, and environmental resistance [1,2,3]. Its strength is derived primarily from coherent γ″ (Ni_3_Nb) and γ′ (Ni_3_(Al,Ti)) precipitates dispersed in the γ matrix [4,5,6]. During take-off, climb, and landing cycles, hot-section components experience cyclic plastic strain arising from coupled thermal and mechanical loading, making high-temperature low-cycle fatigue (LCF) a major life-limiting failure mode [7]. Recent studies on Inconel 718 have shown that high-temperature LCF is commonly characterized by cyclic softening accompanied by planar-slip localization, interactions between dislocations and strengthening precipitates, interfacial damage, and crack initiation and propagation [8,9]. The fatigue response is highly sensitive to mechanical strain amplitude, the initial δ-phase condition, and grain-scale damage localization; increasing strain amplitude intensifies cyclic softening and localized plastic damage, while local deformation near the δ phase and grain boundaries further affects crack initiation and growth [10,11,12]. Accordingly, tailoring the initial microstructure to accommodate changes in the dominant fatigue-damage processes across strain amplitudes remains a key challenge in optimizing the high-temperature LCF resistance of GH4169.

Pre-deformation and subsequent annealing have been employed to tailor the dislocation structure, precipitation behavior, and grain-boundary characteristics of GH4169. Annealing can markedly alter δ-phase precipitation, recovery/recrystallization, and the grain-boundary character distribution [13,14,15,16,17], whereas dislocations and lattice distortion introduced by cold deformation can further affect the precipitation of γ″ and δ phases [18,19,20]. However, pre-deformation does not exert a monotonic effect on fatigue performance. Recent studies have shown that fatigue responses produced by coupled pre-strain–heat-treatment routes and cold working depend strongly on deformation level, subsequent heat treatment, and loading conditions [21,22]. Grain-boundary misorientation and special grain boundaries can also modify slip transfer, local strain concentration, and crack initiation [23,24,25]. Although these studies establish the sensitivity of fatigue to deformation, heat treatment, and grain-boundary characteristics, the effect of adding a 930 °C/10 min anneal after 14% or 20% cold drawing across strain amplitudes remains unresolved. In particular, the coupled roles of deformation substructures, low-angle grain boundaries (LAGBs), and annealing-induced substructure recovery require clarification alongside concurrent precipitation, recrystallization, and residual-stress changes.

Accordingly, the present work examines how short-time annealing changes the strain-amplitude dependence of LCF behavior in cold-drawn GH4169. EBSD analysis, SEM fractography, and TEM characterization of dislocation structures were conducted to examine relationships among the pre-deformation–annealing state, cyclic stress response, fatigue life, and damage features. The working hypothesis was that deformation substructures retained after cold drawing would contribute to local slip accommodation and resistance to crack initiation at low strain amplitude, whereas partial substructure recovery induced by short-time annealing would favor accommodation of larger cyclic plastic strains. Comparisons between matched Z/T conditions at the same drawing level assess whether the relative fatigue-life ranking changes with strain amplitude and whether the accompanying boundary and dislocation observations are consistent with this interpretation.

## 2. Materials and Methods

### 2.1. Material

**Figure 1:**
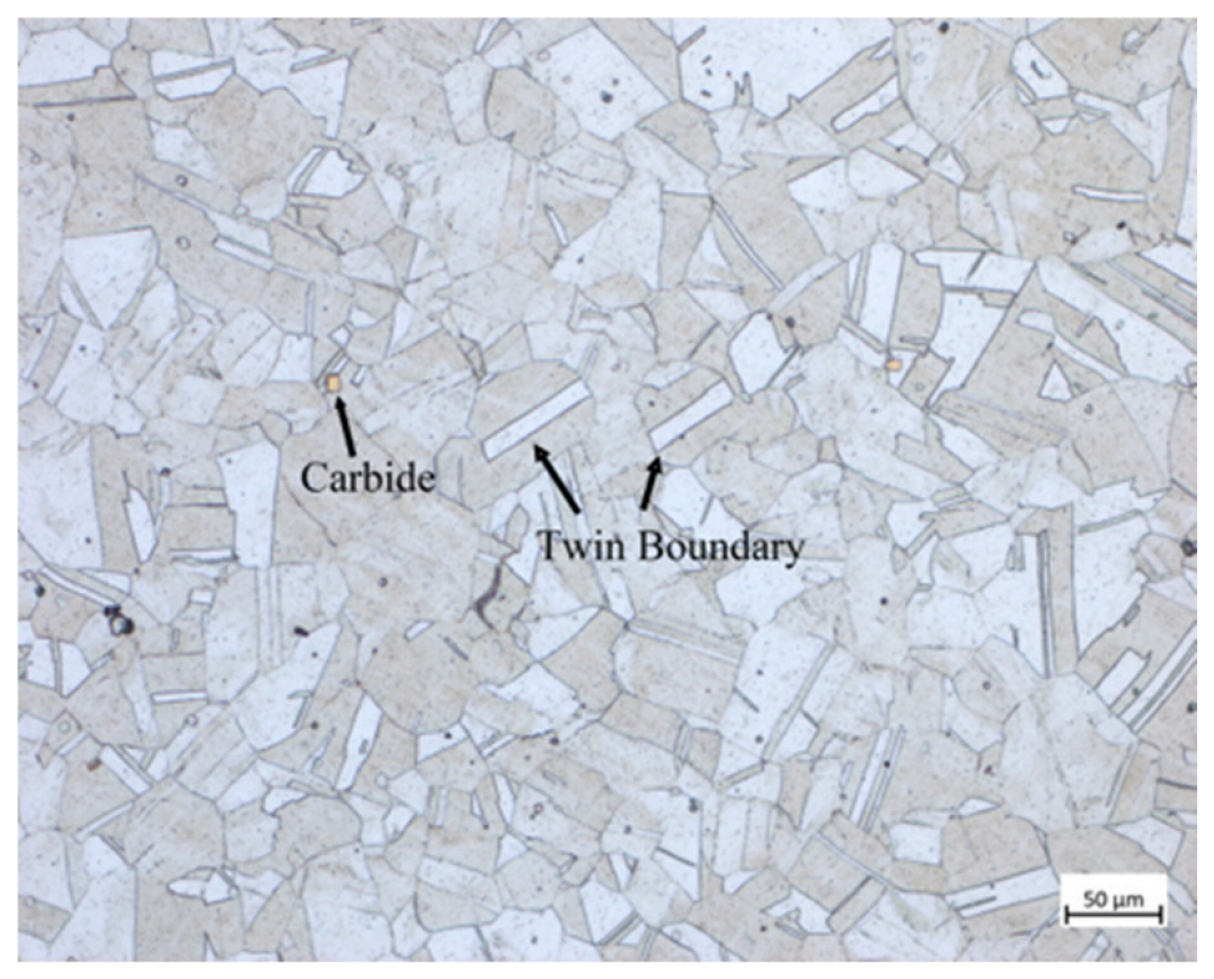
Optical micrograph of the as-received GH4169 alloy.

A forged GH4169 alloy bar produced by vacuum induction melting followed by vacuum arc remelting was used in this study; its chemical composition is listed in Table 1. The as-received material was in the forged condition. After mechanical grinding and polishing, specimens were etched using a solution containing 10 mL HNO_3_, 50 mL HCl, 40 mL H_2_O, and 2.5 g CuCl_2_. The corresponding optical microstructure is shown in Figure 1. The as-received alloy consisted of a γ matrix with relatively straight grain boundaries, numerous intragranular twins, and a small number of carbides retained from the melting and forging process.

To examine fatigue behavior associated with retained cold-deformation substructures and subsequent short-time annealing, a pre-deformation–annealing matrix was designed. The direct-aged condition served as the reference. All other specimens were solution-treated at 980 °C for 30 min and air-cooled, followed by cold drawing at room temperature to diameter reductions of 14% or 20%. Diameter reduction was defined as r = (D_0_ − D_f_)/D_0_, where D_0_ and D_f_ denote the bar diameters before and after drawing. The final diameter was approximately 12 mm for both groups; the initial diameters could not be confirmed from the available processing records. Specimens without the subsequent annealing treatment were denoted 14%-Z and 20%-Z, whereas those annealed at 930 °C for 10 min and air-cooled were denoted 14%-T and 20%-T. All specimens were finally aged at 720 °C for 8 h, furnace-cooled at 50 °C/h to 620 °C, held for 8 h, and then air-cooled. Heat treatment used an XRJX-513 box furnace, with holding time counted after the furnace temperature re-stabilized following specimen insertion. The complete processing routes are summarized in Table 2. Because the direct-aged reference did not undergo the solution treatment, comparisons against it reflect differences in the complete processing routes.

### 2.2. High-Temperature Low-Cycle Fatigue

**Figure 2:**
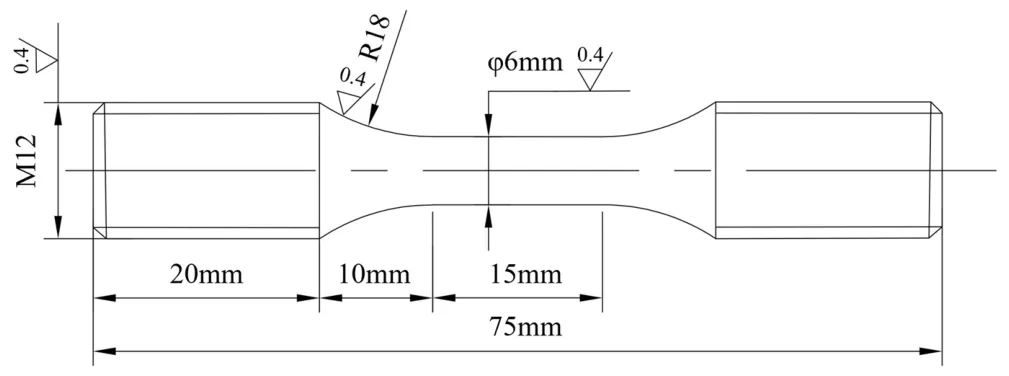
Geometry and dimensions of the high-temperature low-cycle-fatigue specimen.

Specimens were machined and tested under strain control in accordance with GB/T 15248-2008 [26], the Chinese standard for axial constant-amplitude low-cycle fatigue testing of metallic materials. The reduced section had a diameter of 6 mm and a parallel length of 15 mm (Figure 2). The fatigue specimens were machined with their axes parallel to the drawing direction. Before testing, the gauge section was mechanically polished to remove visible machining marks. High-temperature LCF tests were performed on an Instron 8860 fatigue-testing system in air at 650 °C under total-strain control. A fully reversed symmetric triangular waveform was applied with a strain ratio R_ε_ = −1 and a nominal total-strain rate of 0.4% s^−1^. Total strain amplitudes (Δε_t_/2) of 0.4%, 0.6%, and 0.8% were investigated. The corresponding nominal cycle periods were 4, 6, and 8 s, respectively. Total strain was controlled in closed loop using a high-temperature extensometer attached to the gauge section, while load, displacement, and strain signals were recorded continuously. Medium-frequency heating and a K-type thermocouple were used. Specimens were held at 650 °C for 30 min before loading, and the temperature gradient along the gauge section was maintained within 1 °C.

Fatigue failure was defined as either a 50% decrease in the cyclic peak stress from its initial value or complete fracture; the corresponding number of cycles was taken as the fatigue life, N_f_. At 0.4%, the direct-aged and 20%-Z specimens were subsequently loaded in tension to complete fracture after reaching the stress-drop endpoint, so their terminal fracture regions include post-fatigue overload damage. Because specimen-specific endpoints could not be verified for every record, cross-condition life rankings are descriptive and do not establish differences in life to complete fracture. One fatigue-life result is reported for each of the 15 condition–amplitude combinations; independent repeat counts and between-specimen scatter could not be verified.

### 2.3. Microstructural Characterization

The initial microstructures and fatigue-damage features of the different pre-deformation–annealing states were characterized by optical microscopy (OM), scanning electron microscopy (SEM), electron backscatter diffraction (EBSD), and transmission electron microscopy (TEM). Initial-state EBSD was performed on transverse sections at the gauge-section midpoint, perpendicular to the fatigue-specimen axis and the drawing direction. For initial-state EBSD, specimens were mechanically ground and polished and then electropolished at 20 V for approximately 10 s in a solution containing 80 mL methanol and 20 mL sulfuric acid. EBSD measurements were performed using a field-emission SEM equipped with an Oxford EBSD detector at 15 kV, 500×, and a step size of 0.5–1 μm, with analysis in AZtecCrystal (build 5.1.7829.1). Three regions were analyzed for each condition. Boundaries with misorientation angles of 2–15° were classified as LAGBs, whereas those above 15° were classified as high-angle grain boundaries (HAGBs). Σ3 twin boundaries were identified using the approximately 60°/⟨111⟩ misorientation relationship together with the Brandon criterion. Fatigue fracture surfaces were examined using a Prisma E SEM (Thermo Fisher Scientific, Brno, Czech Republic), with particular attention to the crack-initiation, crack-growth, and terminal-fracture regions. Fracture surfaces were ultrasonically cleaned in ethanol and dried before observation; no subsequent mechanical grinding or polishing was performed to preserve the fracture morphology. TEM observations were performed using a Talos F200X G2 microscope (Thermo Fisher Scientific, Eindhoven, The Netherlands) operated at 200 kV. After fatigue testing, foils were sectioned approximately 2 mm from the fracture surface, normal to the loading axis. The foils were mechanically thinned to approximately 50 μm, punched into 3 mm disks, and twin-jet electropolished at −25 °C and 20 V using a 10% perchloric acid–ethanol solution.

## 3. Results

### 3.1. Grain and Grain-Boundary Characteristics

**Figure 3:**
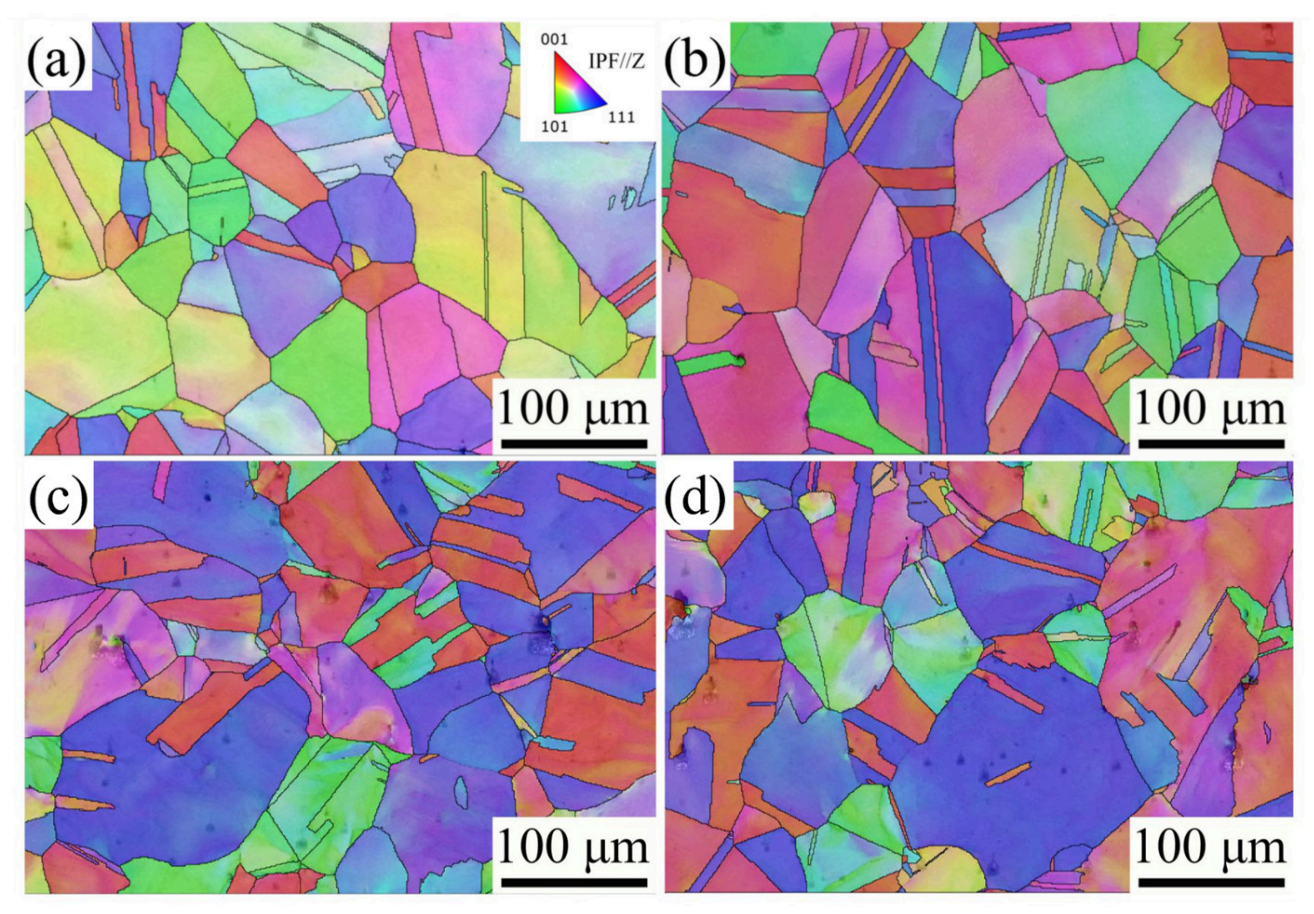
IPF maps of specimens subjected to different cold-drawing pre-deformation levels: (**a**) 14%-Z; (**b**) 14%-T; (**c**) 20%-Z; and (**d**) 20%-T.

**Figure 4:**
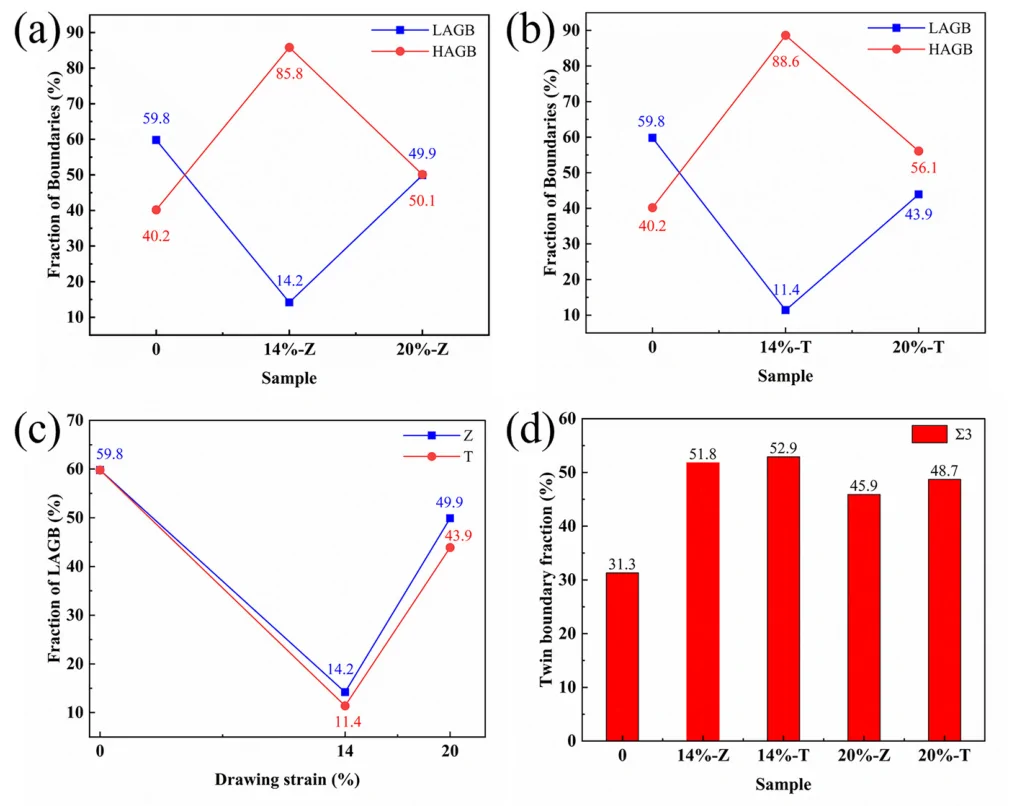
Fractions of LAGBs, HAGBs, and Σ3 twin boundaries: (**a**) Z series; (**b**) T series; (**c**) comparison between Z and T states; and (**d**) Σ3 twin boundaries.

Figure 3 presents inverse pole figure (IPF) maps of specimens subjected to 14% and 20% cold-drawing pre-deformation with and without the subsequent short-time annealing treatment. All conditions were dominated by equiaxed grains with well-defined grain boundaries and numerous intragranular twins, while the overall grain morphology remained similar among the cold-drawn conditions. Mean grain sizes were 20.90 μm for the direct-aged reference, 31.17 and 30.71 μm for 14%-Z and 14%-T, and 35.14 and 38.07 μm for 20%-Z and 20%-T (Table 3). These grain-size differences may reflect solution treatment and deformation-dependent boundary migration during subsequent heat treatment. Increasing the cold-drawing pre-deformation from 14% to 20% produced a more pronounced change in the local orientation distribution. The fractions of LAGBs, HAGBs, and Σ3 twin boundaries are summarized in Figure 4. For the Z conditions, the LAGB fraction increased markedly from 14.2% at 14% pre-deformation to 49.9% at 20%; for the T conditions, it increased from 11.4% to 43.9%, accompanied by a corresponding decrease in HAGBs. This increase is consistent with changes in dislocation accumulation and rearrangement at the higher cold-drawing level. At a given pre-deformation level, short-time annealing at 930 °C for 10 min reduced the LAGB fraction from 14.2% to 11.4% for the 14% condition and from 49.9% to 43.9% for the 20% condition, consistent with partial recovery of the cold-deformation substructure.

Mean kernel average misorientation (KAM) values were 1.051° for the direct-aged reference, 0.404° and 0.428° for 14%-Z and 14%-T, and 0.729° and 0.685° for 20%-Z and 20%-T (Table 3). The lower value after annealing at 20% drawing is consistent with reduced local misorientation, whereas the 14% pair shows a small increase. The KAM response therefore differs between the two drawing levels; these values describe local orientation gradients rather than directly measuring dislocation density.

The Σ3 twin-boundary fraction exhibited a different trend from that of the LAGBs. Increasing the cold-drawing pre-deformation from 14% to 20% decreased the Σ3 fraction from 51.8% to 45.9% in the Z series and from 52.9% to 48.7% in the T series. By contrast, the influence of short-time annealing on the Σ3 fraction was limited: it remained nearly unchanged at 14% pre-deformation and increased only slightly, from 45.9% to 48.7%, at 20%. Thus, increasing the cold-drawing pre-deformation within the Z and T series primarily increased the LAGB fraction while decreasing the Σ3 fraction, whereas short-time annealing was associated with lower LAGB fractions. The direct-aged reference had a higher LAGB fraction of 59.8% and an HAGB fraction of 40.2%. Its different thermal history, without the solution treatment applied to the cold-drawn states, means that this reference does not establish a monotonic cold-drawing effect across all five conditions. Its higher LAGB fraction and mean KAM may also reflect deformation inherited from forging. The higher LAGB fraction of the reference did not give the highest recorded N_f_ at 0.4%, indicating that LAGB fraction alone cannot explain the life ranking.

### 3.2. Fatigue Life

**Figure 5:**
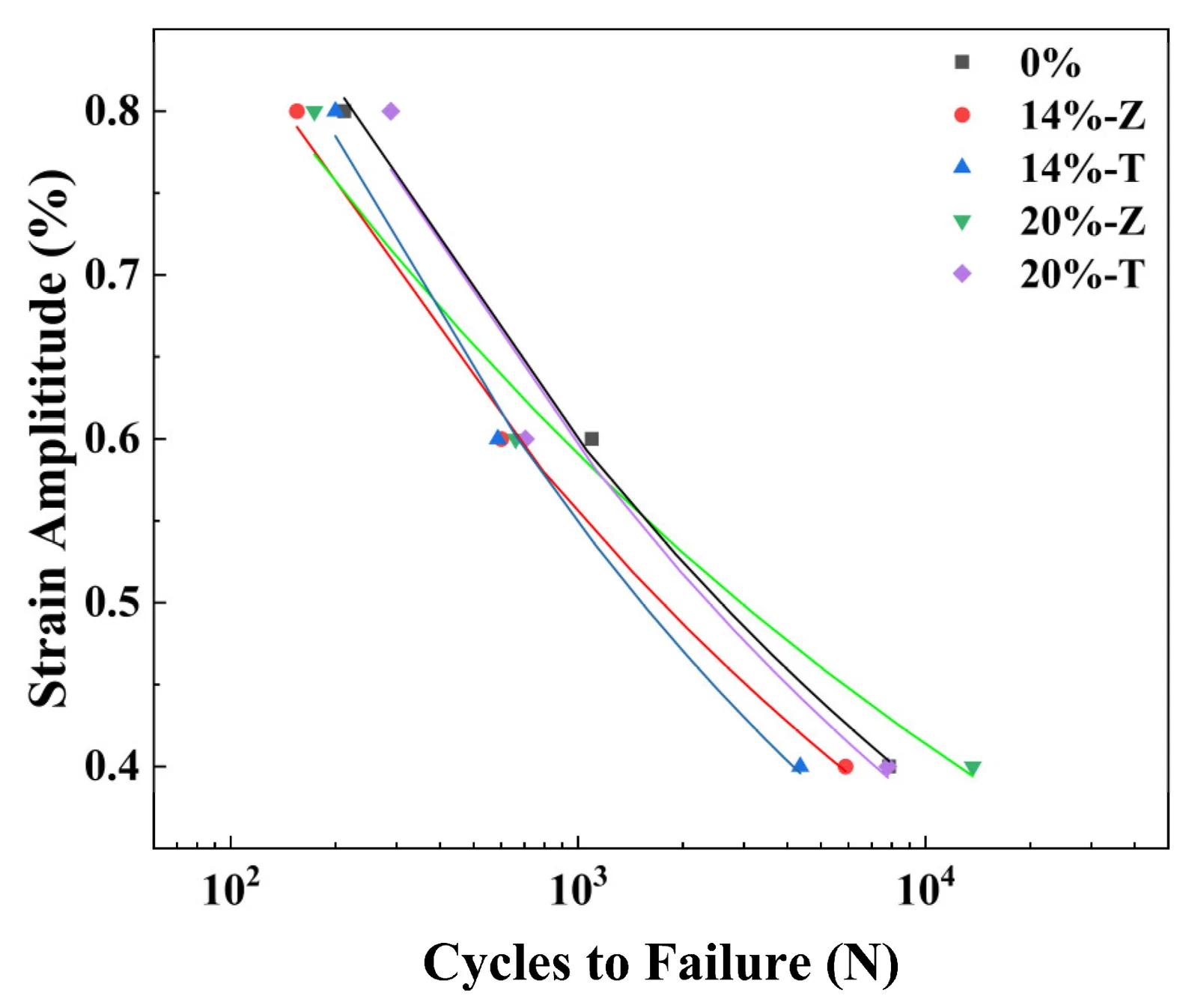
Strain–life curves for specimens with different pre-deformation–annealing states.

Figure 5 and Table 4 show the strain–life relationships of GH4169 under the different pre-deformation–annealing states at 650 °C. Fatigue life decreased markedly with increasing total strain amplitude for all conditions, but the relative ranking of the conditions changed with strain amplitude. At Δε_t_/2 = 0.4%, 20%-Z exhibited the highest recorded N_f_, reaching 13,679 cycles, and for a given cold-drawing level the unannealed Z condition had a longer life than the corresponding T condition. At 0.6%, the life differences among the cold-drawn conditions narrowed substantially, while the direct-aged reference exhibited the highest recorded N_f_ of 1095 cycles. When the strain amplitude was further increased to 0.8%, 20%-T exhibited the highest recorded N_f_ of 289 cycles, and the T conditions had longer lives than their corresponding Z conditions.

A direct comparison between the Z and T conditions at the same cold-drawing level further demonstrates the strain-amplitude dependence of the annealing response. At the low strain amplitude of 0.4%, both 14%-Z and 20%-Z exhibited longer lives than their corresponding T conditions, with a particularly pronounced difference at 20% pre-deformation. At 0.6%, 20%-T already had a slightly longer life than 20%-Z (705 versus 661 cycles), whereas 14%-Z still exceeded 14%-T (601 versus 587 cycles); at 0.8%, both T conditions had longer lives than their corresponding Z conditions. These results suggest that retaining the deformation substructure can be beneficial at low strain amplitude, whereas short-time annealing may become favorable as the cyclic strain level increases. However, the longest life of the direct-aged reference at 0.6% shows that this is a crossover in the matched Z/T comparisons, rather than a universal treatment ranking. Thus, the influence of cold-drawing pre-deformation and short-time annealing on LCF life depends on strain amplitude within the investigated range.

### 3.3. Cyclic Hysteresis Behavior and Stress Response

**Figure 6:**
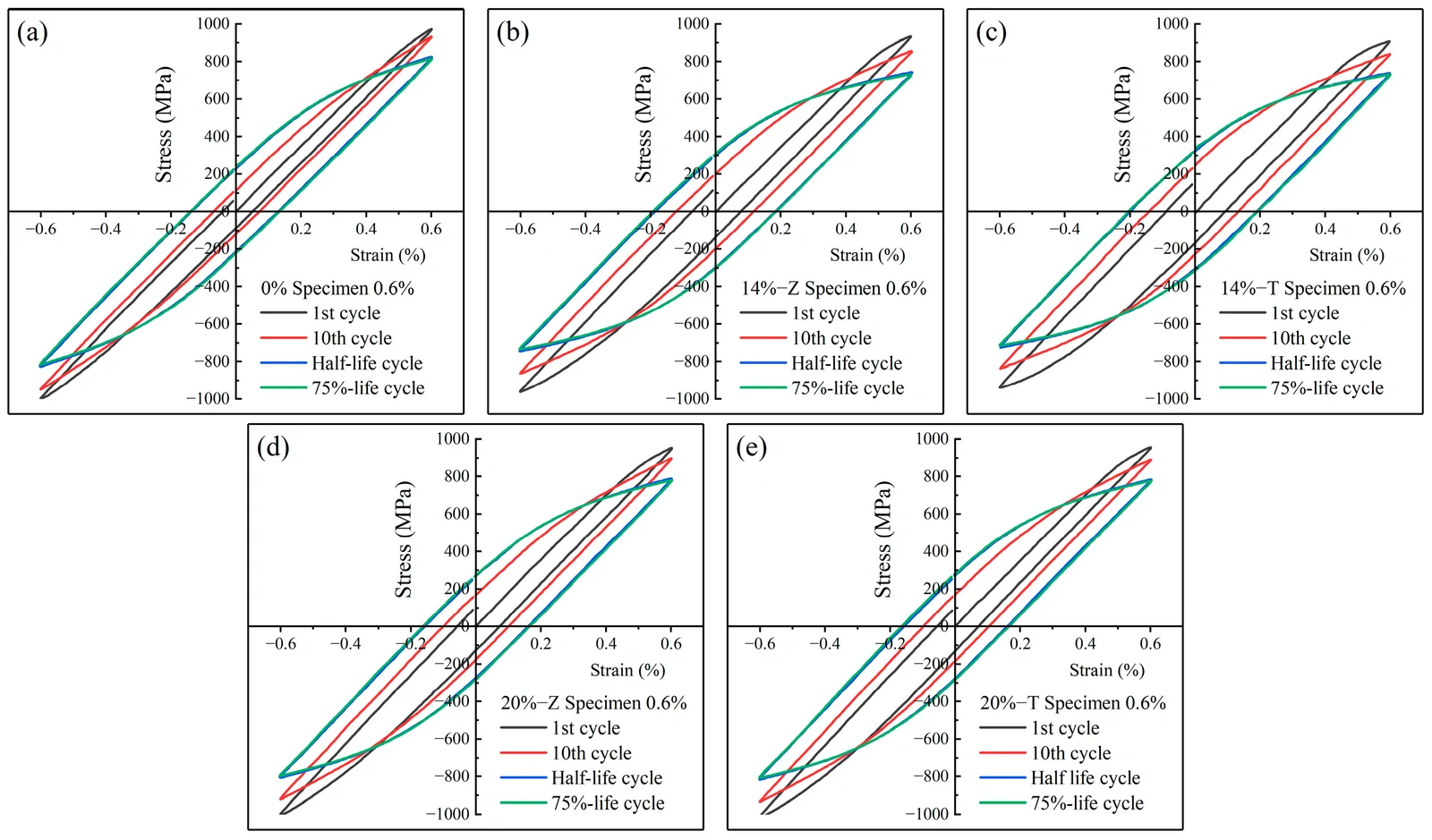
Cyclic hysteresis loops of specimens with different pre-deformation–annealing states at Δε_t_/2 = 0.6%: (**a**) 0%; (**b**) 14%-Z; (**c**) 14%-T; (**d**) 20%-Z; and (**e**) 20%-T.

Figure 6 shows the hysteresis loops of the different conditions at Δε_t_/2 = 0.6% for the first cycle, the 10th cycle, half-life, and 75% of fatigue life. The overall stress level decreased with cycling for all conditions, indicating cyclic softening at this intermediate strain amplitude, although the cyclic response depended on the initial microstructural state. The direct-aged reference maintained a relatively high stress level throughout cycling and exhibited comparatively narrow loops. The 14%-Z and 14%-T conditions showed lower stress levels and wider loops, with 14%-T exhibiting a larger plastic strain component. The responses of 20%-Z and 20%-T were intermediate and relatively similar to each other. This trend is consistent with the fatigue-life ranking at 0.6%: the direct-aged reference showed the longest life, whereas the lives of the 14% and 20% cold-drawn conditions were comparatively close. The results suggest that, at the intermediate strain amplitude, fatigue behavior is associated with both cyclic deformation resistance and plastic-strain partitioning. Half-life and 75%-life comparisons correspond to the same relative life fractions but different absolute cycle counts and exposure times. The reported half-life plastic strain amplitudes for all conditions are listed in Table 5.

**Figure 7:**
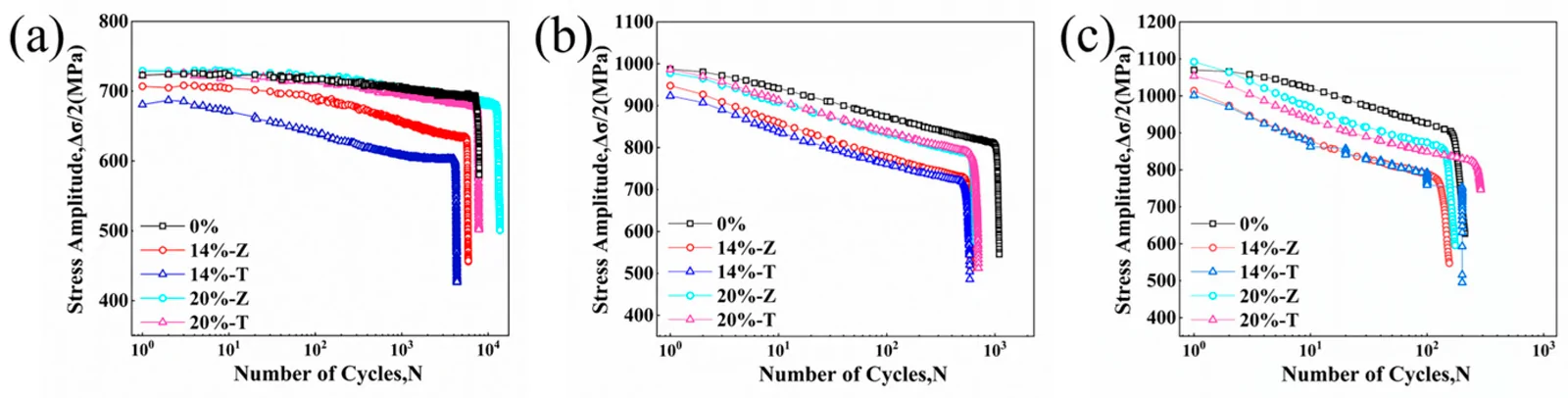
Cyclic stress response curves at total strain amplitudes of (**a**) 0.4%, (**b**) 0.6%, and (**c**) 0.8%.

Figure 7 compares the cyclic stress responses at different strain amplitudes. All conditions were dominated by cyclic softening. As the total strain amplitude increased from 0.4% to 0.8%, the initial stress amplitude increased and the slowly softening regime became shorter. Such cyclic softening is consistent with previous observations for GH4169 and is commonly associated with dislocation-structure rearrangement, repeated interactions between dislocations and strengthening precipitates, and progressive fatigue damage [27,28,29]. The effect of the pre-deformation–annealing state was likewise strain-amplitude dependent. The 14%-Z and 14%-T specimens exhibited sustained softening at all three amplitudes and only limited differences in cyclic response. By contrast, the Z/T difference was more pronounced after 20% cold drawing. At 0.4%, 20%-Z exhibited an extended slowly softening regime; this regime shortened markedly as the strain amplitude increased. Although 20%-T showed a less sustained slowly softening response than 20%-Z at low strain amplitude, its terminal stress decrease extended over more cycles at 0.8%. These qualitative trends are consistent with the observed life advantage of 20%-Z at 0.4% and 20%-T at 0.8%, illustrating the strain-amplitude dependence of the cyclic response.

### 3.4. Fatigue Fractography of Low Strain Amplitude

**Figure 8:**
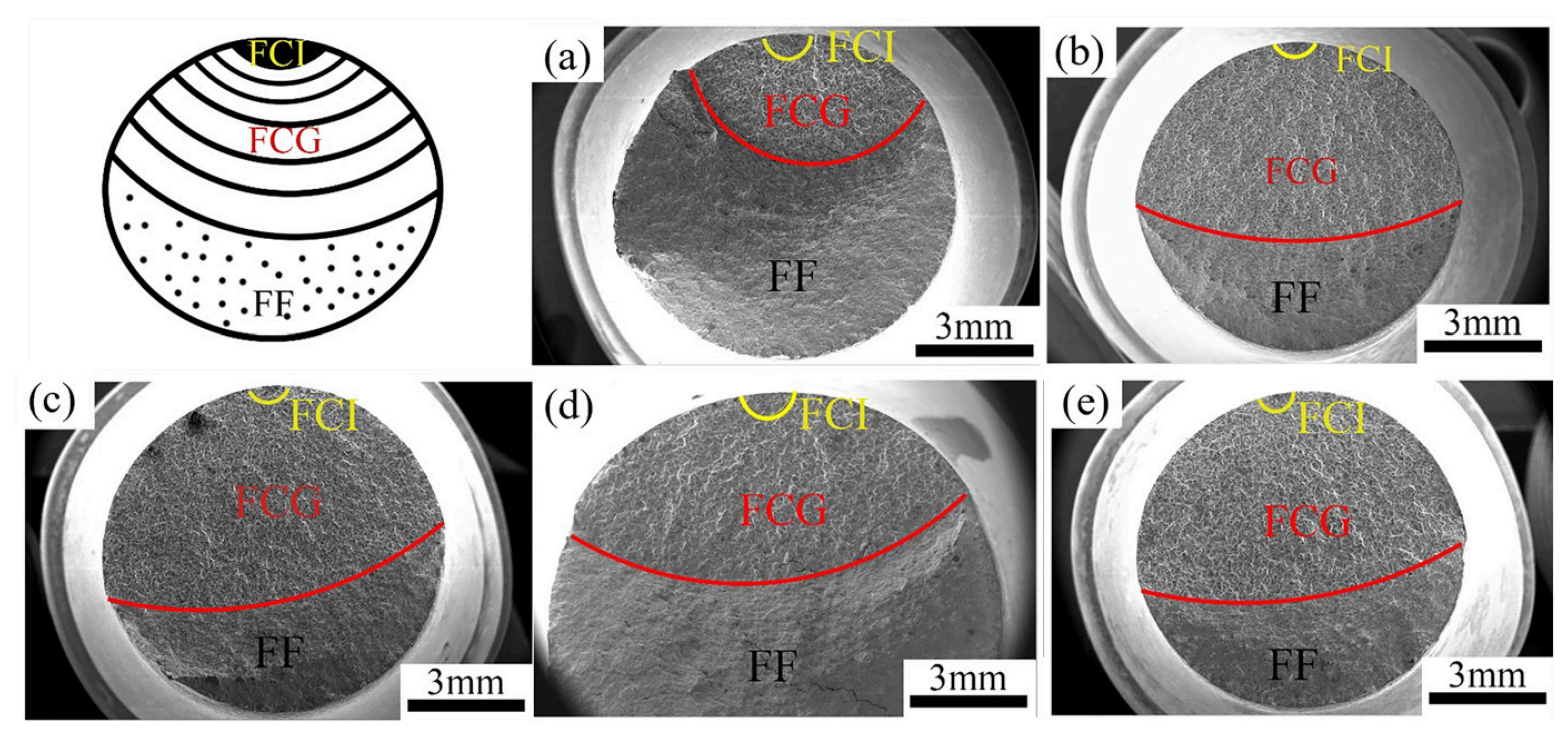
Macroscopic fatigue-fracture morphologies of GH4169 at Δε_t_/2 = 0.4%: (**a**) 0%; (**b**) 14%-Z; (**c**) 14%-T; (**d**) 20%-Z; and (**e**) 20%-T.

**Figure 9:**
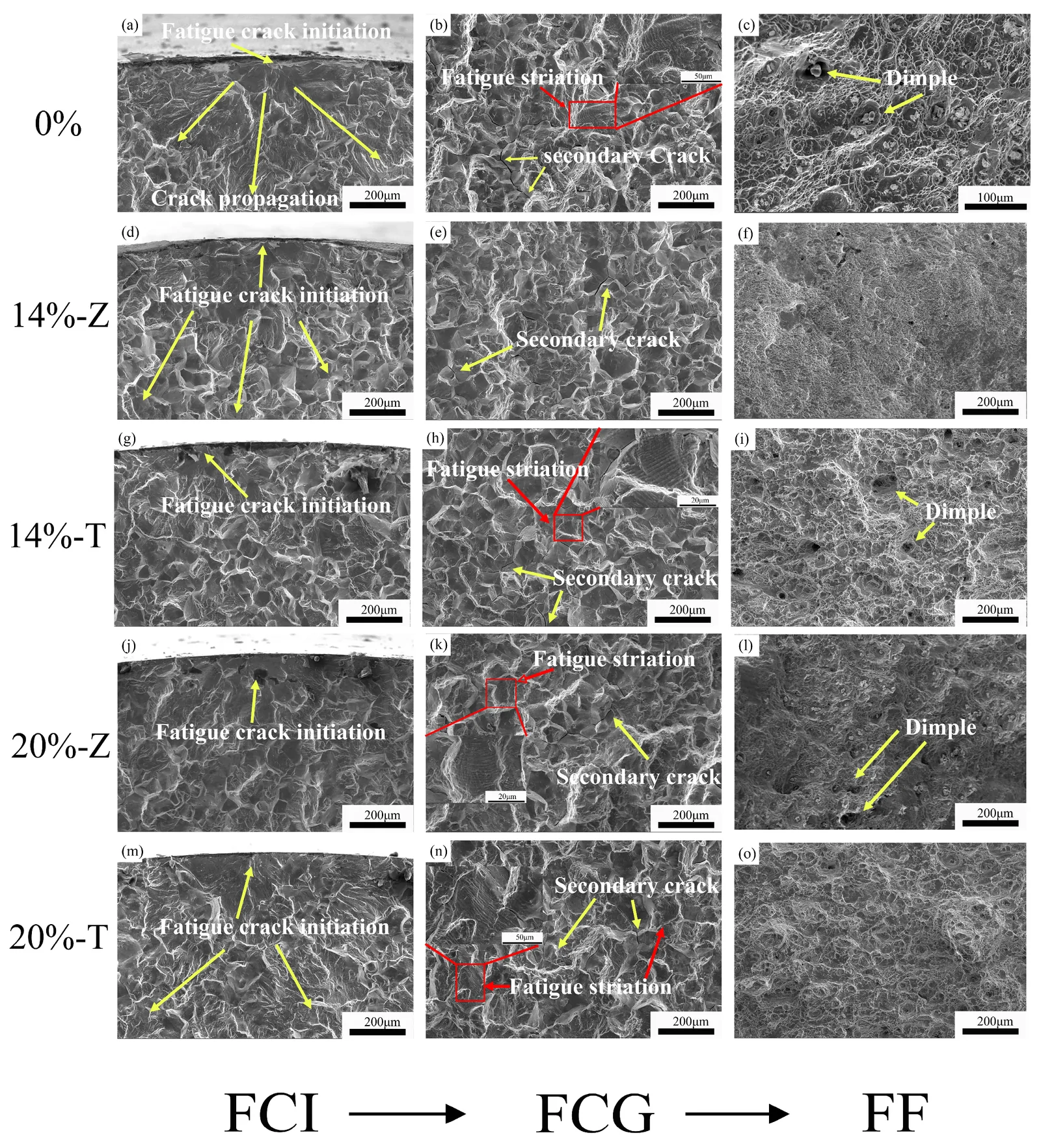
Fracture morphologies at Δε_t_/2 = 0.4%: (**a**–**c**) 0%; (**d**–**f**) 14%-Z; (**g**–**i**) 14%-T; (**j**–**l**) 20%-Z; and (**m**–**o**) 20%-T. The columns show the inferred FCI, FCG, and FF regions, respectively.

Figure 8 and Figure 9 show the macroscopic and microscopic fatigue-fracture morphologies, respectively, of the different GH4169 conditions tested at Δε_t_/2 = 0.4%. All specimens exhibited typical fatigue-fracture features, with an inferred fatigue crack initiation (FCI) region, a fatigue crack growth (FCG) region, and a final fracture (FF) region. The images suggest predominantly surface-associated origins and inward propagation under the low-strain-amplitude condition. The relative extents of the FCG and FF regions varied among the conditions. However, the direct-aged and 20%-Z specimens were subsequently loaded in tension to complete fracture after reaching the stress-drop endpoint. Their FF regions therefore include post-fatigue overload, and the apparent FCG area is not an independent measure of fatigue life.

Figure 9 presents higher-magnification morphologies of three representative regions of the fracture surfaces. In the direct-aged specimen, clear fatigue striations and secondary cracks were observed in the FCG region, whereas the FF region was dominated by dimples and microvoid coalescence. The 14%-T condition exhibited comparatively continuous fatigue striations in the FCG region, while 14%-Z showed more evident local intergranular propagation features together with secondary cracks. Typical fatigue-growth features were also observed in the FCG regions of both 20%-Z and 20%-T, and their FF regions remained dominated by dimples and microvoid coalescence. Overall, the representative surfaces suggest surface crack initiation and mixed intergranular/transgranular propagation at 0.4%, followed by predominantly ductile final fracture. These differences indicate that the pre-deformation–annealing state affects the crack-growth morphology; nevertheless, final-fracture surfaces alone cannot quantitatively separate the contributions of crack initiation and stable crack growth to total fatigue life [25].

The fractographic differences are consistent with an effect of the pre-deformation–annealing state on fatigue-crack propagation; however, the lifetime advantage of 20%-Z at 0.4% cannot be attributed to crack-growth resistance on the basis of the final fracture surface alone. The more pronounced local intergranular features observed in 14%-Z may indicate a greater tendency for grain-boundary-assisted propagation. Because fractography primarily records the propagation and final-fracture stages, the proposed crack-initiation mechanism is considered together with the grain-boundary characteristics and TEM observations near the fracture surface [25,30,31].

## 4. Discussion

### 4.1. Fatigue Damage and Fracture Mechanisms

**Figure 10:**
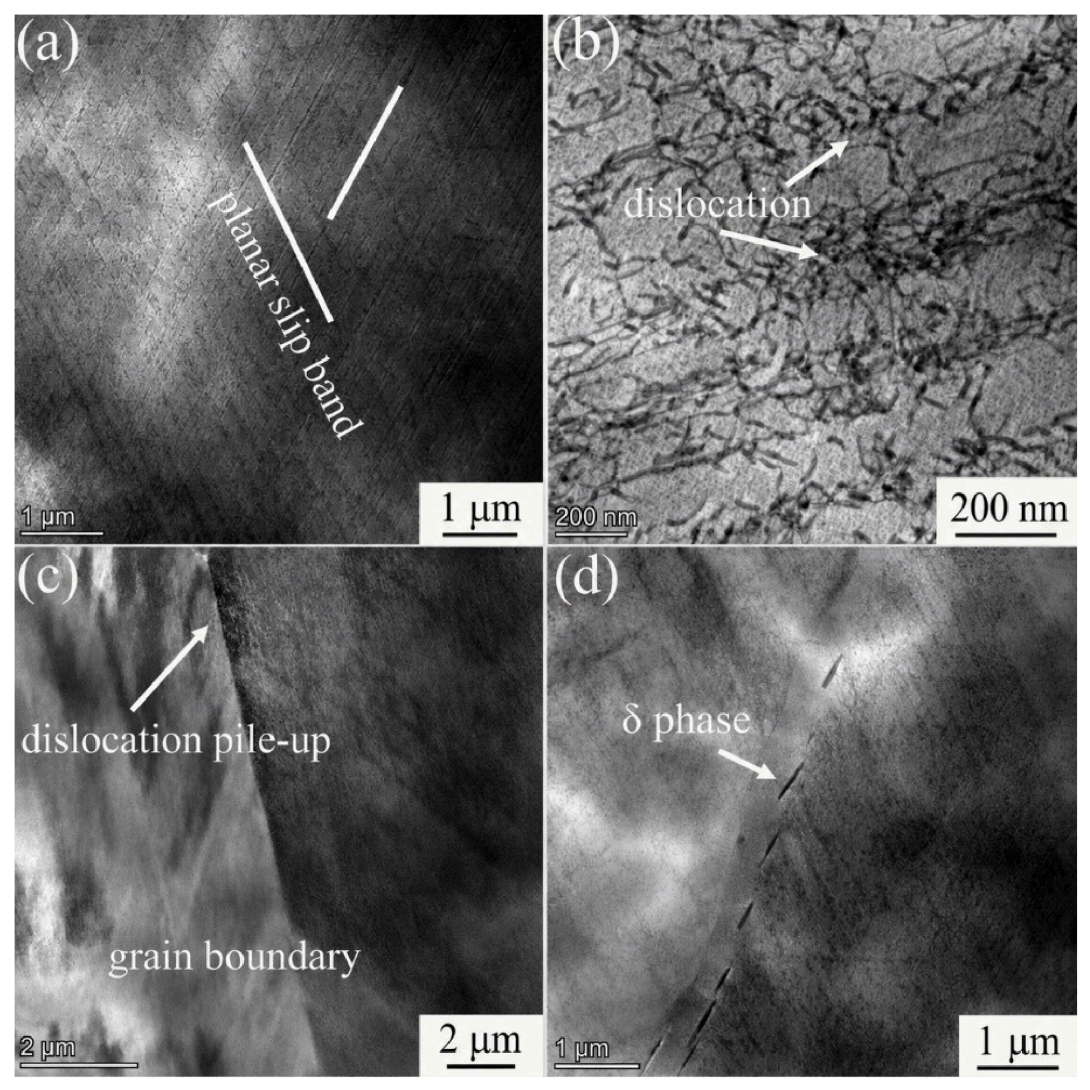
TEM microstructures near the fatigue fracture of 20%-Z at 650 °C and Δε_t_/2 = 0.4% (N_f_ = 13,679): (**a**) planar slip bands; (**b**) dislocation morphology; (**c**) grain-boundary pile-up; and (**d**) pile-up near a particle tentatively identified as δ phase.

**Figure 11:**
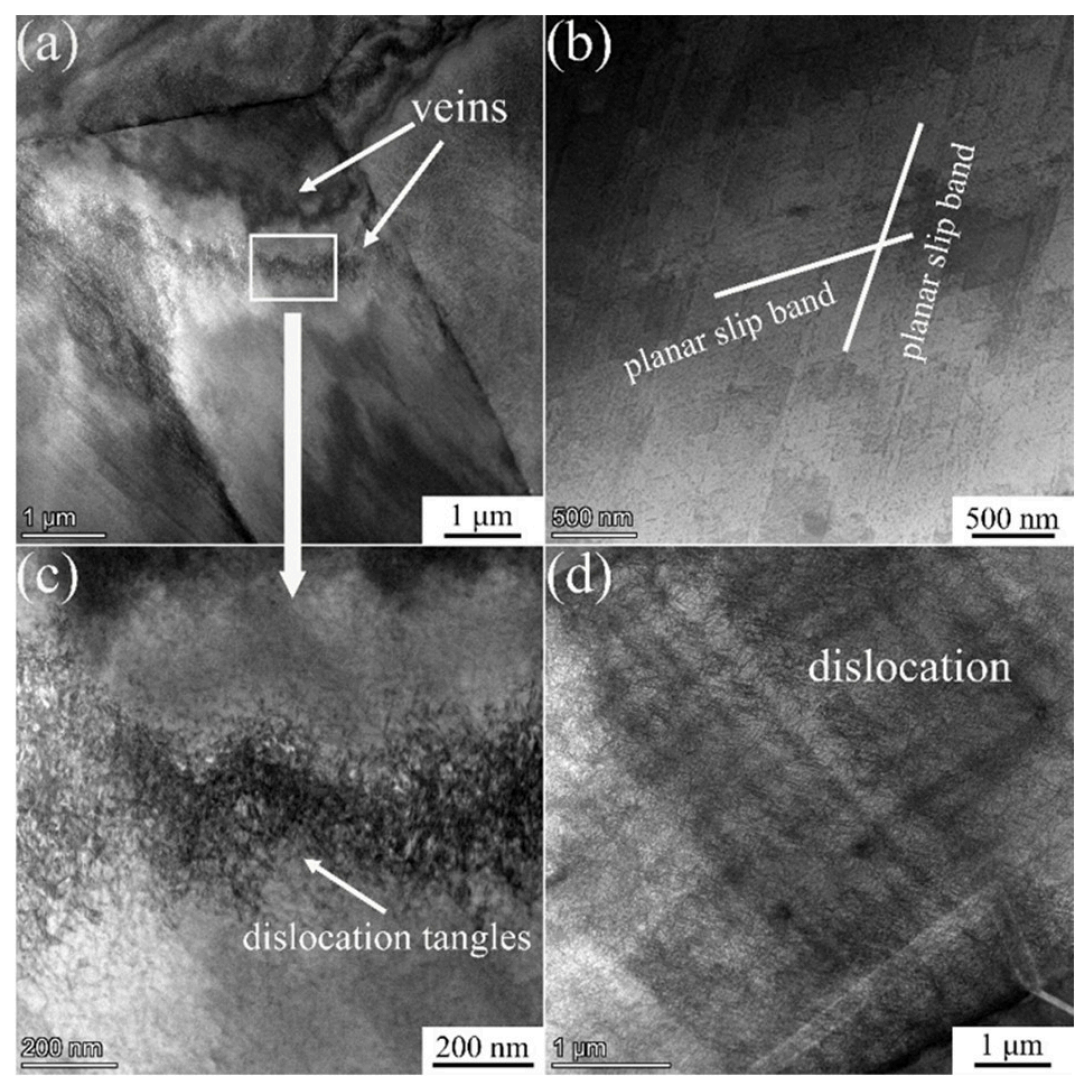
TEM dislocation structures near the fatigue fracture of the 20%-Z specimen tested at 650 °C and Δε_t_/2 = 0.6% (N_f_ = 661): (**a**) dislocation veins; (**b**) planar slip bands; (**c**) dislocation tangles; and (**d**) high-density dislocation structures.

Figure 10 and Figure 11 show selected 20%-Z fields after testing at 0.4% and 0.6%, respectively, with attention to slip bands, dislocation pile-ups, and interfacial interactions. The particle in Figure 10d is tentatively assigned to δ by morphology. At low strain amplitude, the selected 20%-Z microstructures were characterized by localized planar slip. Figure 10 shows the near-fracture TEM microstructure of the 20%-Z specimen after testing at 650 °C and Δε_t_/2 = 0.4%, for which N_f_ = 13,679 cycles. Directional planar slip bands and localized dislocation structures were observed within the grains. Pronounced pile-ups were present near grain boundaries and a particle tentatively assigned to δ based on its morphology. These observations suggest that, at the lower cyclic-plasticity level, fatigue damage involves irreversible dislocation motion within a limited number of slip bands and its progressive accumulation near interfaces. The resistance of grain boundaries and second-phase particles to slip transfer can increase local lattice curvature, dislocation storage, and plastic-strain gradients; continued accumulation of such incompatibility during cycling can create preferential sites for crack initiation [25,30,31].

The LAGB fraction of 20%-Z reached 49.9%, consistent with a substantial deformation substructure after 20% cold-drawing pre-deformation. Low-angle boundaries with relatively small misorientations may provide greater geometric compatibility for slip transfer, while the deformation substructure supplies additional interfaces for dislocation storage and rearrangement [23,25]. Such a substructure could help distribute cyclic plasticity and reduce excessive slip localization near selected grain boundaries. The 20%-Z condition may therefore combine dislocation constraint with a capacity for accommodating local slip. Together with its extended slowly softening regime and longest life at 0.4%, these observations support a possible contribution of the retained deformation substructure to low-strain-amplitude fatigue resistance.

When the total strain amplitude was increased to 0.6%, the fatigue life of the same 20%-Z initial condition decreased to 661 cycles, approximately one-twentieth of that at 0.4%. As shown in Figure 11, vein-like dislocation structures and dense dislocation tangles were observed near grain boundaries, while planar slip remained evident within the grains. The selected fields at 0.6% contained more extensive tangles and vein-like structures than those at 0.4%, suggesting stronger dislocation interaction and rearrangement. Because both TEM observations were obtained near the fracture surfaces after testing, these structures reflect both the imposed amplitude and the very different accumulated cycle histories. Dislocation accumulation near grain boundaries can increase local plastic incompatibility, whereas intragranular planar-slip bands provide paths for irreversible plastic strain. These observations are consistent with more extensive localized cyclic plasticity in the examined 0.6% fields and the shorter slowly softening regime of 20%-Z.

**Figure 12:**
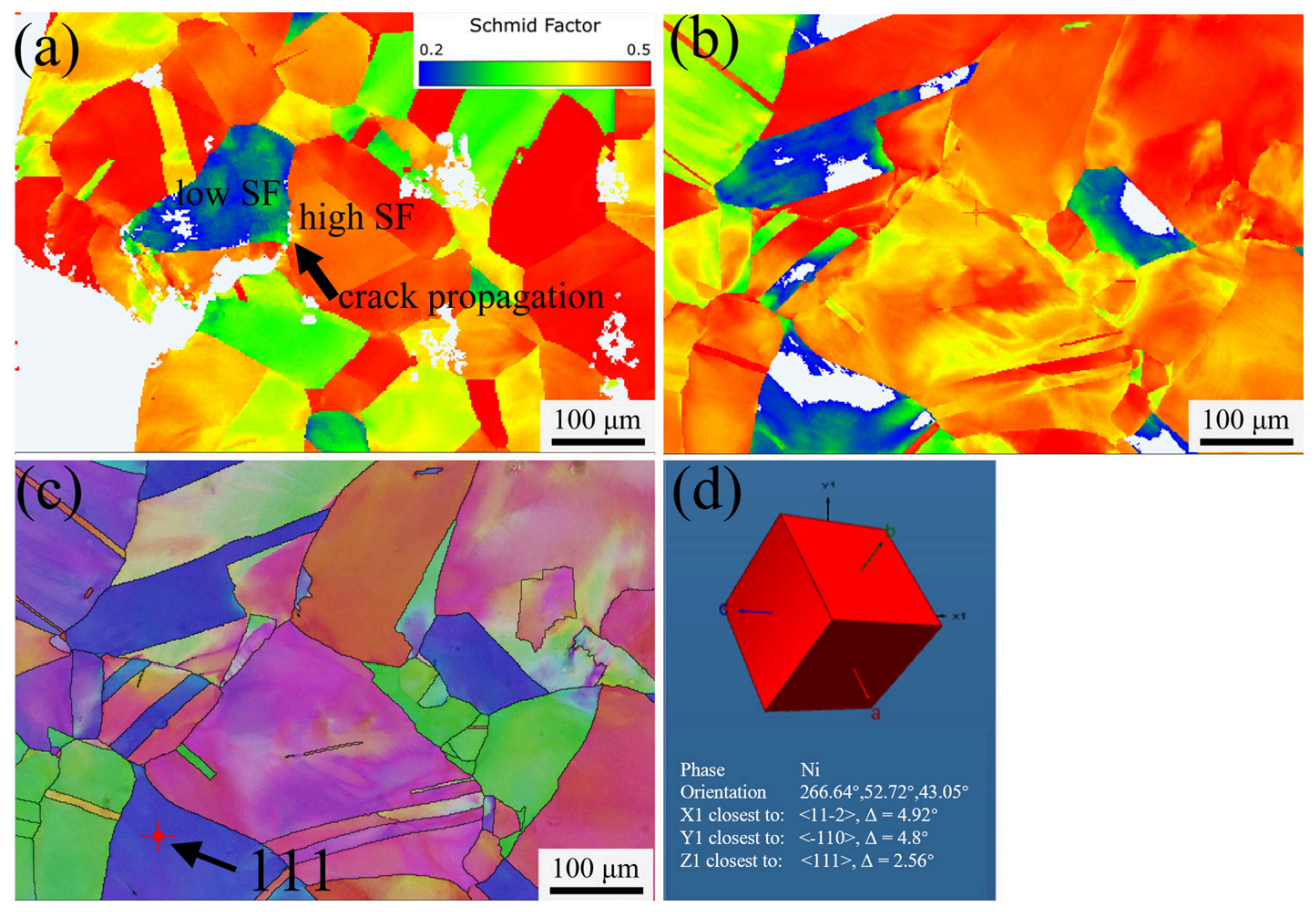
Crystallographic analysis of a representative crack-growth region: (**a**,**b**) Schmid-factor maps; (**c**) corresponding IPF map; and (**d**) (111) orientation. The black arrow in (**a**) indicates crack propagation, while the black arrow and red cross in (**c**) mark the grain whose orientation is shown in (**d**).

In addition to the cyclic dislocation structure, the path of an established crack is influenced by local crystallographic orientation and intergranular slip compatibility. For the face-centered-cubic γ matrix of GH4169, the primary slip systems are {111}⟨110⟩. The Schmid-factor maps in Figure 12 qualitatively illustrate variations in slip-activation tendency along a representative crack path. In general, grains with higher Schmid factors experience larger resolved shear stresses under the assumed loading reference and activate slip more readily, promoting continuous slip bands that can provide transgranular crack paths. In grains with lower Schmid factors, or at interfaces between neighboring grains with markedly different slip activity, discontinuous slip transfer can increase local plastic incompatibility. The crack path is therefore influenced by the combined effects of slip activation in adjacent grains, grain-boundary geometric compatibility, and the local plastic field at the crack tip [25,30]. The ⟨111⟩ illustration in Figure 12d denotes orientation, whereas the 60°/⟨111⟩ Σ3 relationship describes twin misorientation; neither denotes the ⟨110⟩ slip direction.

**Figure 13:**
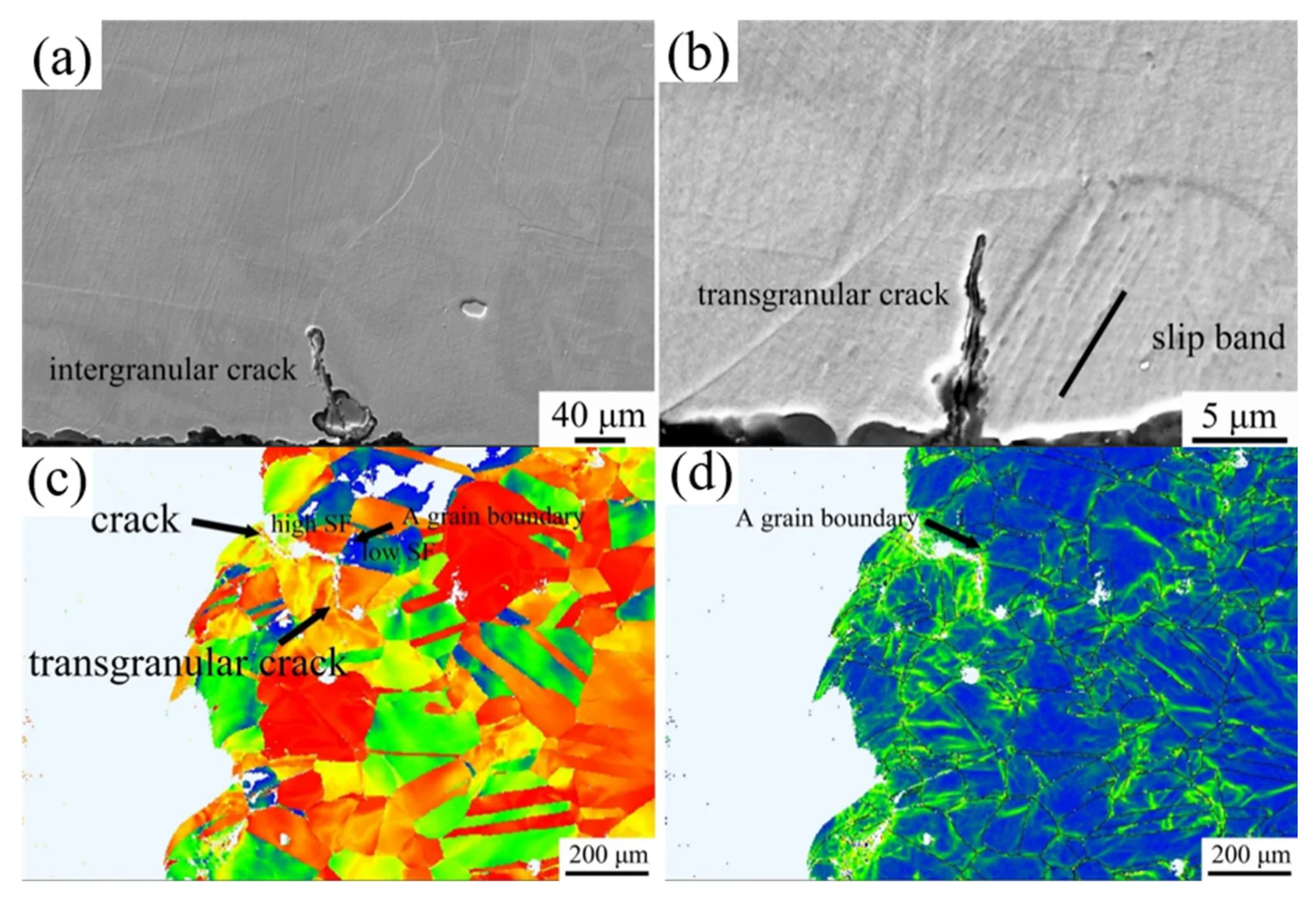
Crack propagation and local plastic deformation in a representative fatigue-damaged region after failure: (**a**) intergranular crack; (**b**) transgranular crack propagating along slip bands; (**c**) crack deflection at a grain boundary and the corresponding Schmid-factor distribution; and (**d**) corresponding KAM map.

Figure 13 further illustrates the coupled roles of intragranular slip, grain-boundary constraint, and crack-path selection. In Figure 13a, a region adjacent to a grain with no obvious slip bands shows intergranular cracking, whereas Figure 13b shows a clear transgranular crack developing along a slip band, suggesting that intragranular slip influences the preferred propagation path. Figure 13c,d further show that the main crack was deflected at a specific grain boundary before re-entering the grain interior. The corresponding KAM map exhibits locally elevated misorientation near the boundary and crack tip, consistent with local lattice curvature and heterogeneous deformation. Grain boundaries can therefore play a dual role during fatigue-crack propagation: slip incompatibility across a boundary can favor local strain concentration, whereas resistance to slip transfer can promote crack deflection or an alternative transgranular path [25,30,31].

Taken together, Figure 10, Figure 11, Figure 12 and Figure 13 highlight the coupling between cyclic-plasticity localization and intergranular deformation compatibility during high-temperature LCF of GH4169. At low strain amplitude, LAGBs and deformation substructures retained after cold drawing may facilitate dislocation storage and slip redistribution, limiting excessive localization near individual interfaces. The more extensive tangles and vein-like structures observed at 0.6% are consistent with increased local dislocation interaction, although their development also reflects the accumulated fatigue history. Once a crack forms, its propagation path is influenced by local slip activation, grain-orientation differences, grain-boundary compatibility, and the plastic state at the crack tip.

### 4.2. Half-Life Cyclic Plastic Response

To further correlate cyclic plastic response with fatigue life, the half-life hysteresis loops were compared with the reported plastic strain amplitudes. The enclosed loop area reflects bulk per-cycle dissipation [32,33,34,35,36,37,38]; here, loop shapes and plastic strain amplitudes are compared qualitatively.

**Figure 14:**
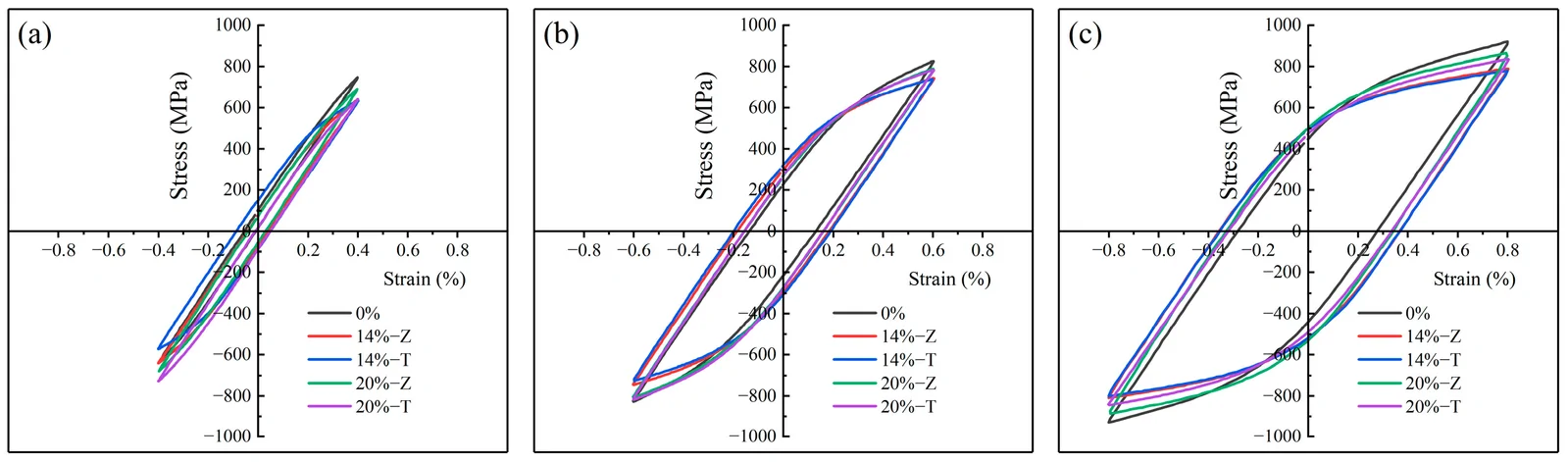
Half-life hysteresis loops of specimens with different pre-deformation–annealing states at total strain amplitudes of (**a**) 0.4%, (**b**) 0.6%, and (**c**) 0.8%.

As shown in Figure 14, the hysteresis loops broadened markedly as the total strain amplitude increased from 0.4% to 0.8%, accompanied by an increase in the plastic strain component. The reported half-life plastic strain amplitudes for all 15 combinations are given in Table 5. At 0.4%, 20%-Z exhibited a plastic strain amplitude of 0.0346% and a fatigue life of 13,679 cycles. By contrast, 14%-T showed a larger plastic strain amplitude of 0.0684%, and its fatigue life was 4360 cycles. Combined with the low-strain-amplitude TEM observations and grain-boundary statistics, the lower bulk plastic strain of 20%-Z is consistent with a contribution from its retained deformation substructure. However, 20%-T had a still smaller plastic amplitude of 0.0298% and a shorter life of 7794 cycles, showing that bulk strain partitioning alone does not explain the life ranking.

At Δε_t_/2 = 0.6%, the half-life plastic strain amplitudes of 20%-Z and 20%-T were 0.1585% and 0.1580%, respectively, and their fatigue lives were 661 and 705 cycles. Their macroscopic cyclic responses were therefore very similar. Meanwhile, the TEM comparison for the same 20%-Z condition in Section 4.1 showed more extensive dislocation tangling and vein formation at 0.6% than at 0.4%, consistent with stronger localized plastic activity in the examined fields. At this intermediate amplitude, the low-amplitude life advantage of the unannealed condition had largely diminished. The direct-aged reference nevertheless had a smaller plastic strain amplitude of 0.1336% and the longest observed life of 1095 cycles, emphasizing the influence of its distinct initial state.

When the strain amplitude was further increased to 0.8%, the half-life plastic strain amplitudes of 20%-Z and 20%-T were 0.3353% and 0.3276%, respectively. Despite these similar macroscopic cyclic-plastic responses, the observed fatigue life increased from 174 cycles for 20%-Z to 289 cycles for 20%-T. The direct-aged reference exhibited a smaller plastic amplitude of 0.2795% but a life of 212 cycles, again indicating that bulk plastic strain alone does not govern fatigue resistance. Short-time annealing reduced the LAGB fraction of the 20% cold-drawn material from 49.9% to 43.9%, consistent with partial recovery of the deformation substructure. Such recovery may reduce excessive local constraint and improve cyclic plastic-strain accommodation at high amplitude, providing a possible explanation for the longer life of 20%-T.

Overall, the grain-boundary characteristics, cyclic hysteresis response, and half-life plastic strain amplitudes suggest a strain-amplitude-dependent balance between constraint and accommodation of cyclic plasticity. At low strain amplitude, the deformation substructure retained in 20%-Z may help distribute slip and limit excessive plastic localization. As strain amplitude increases, more extensive dislocation interaction and local plastic incompatibility can increase the importance of accommodating larger cyclic plastic strains. Partial substructure recovery after annealing offers a plausible contribution to this accommodation and to the reversal of the observed 20%-Z/20%-T life ranking.

### 4.3. Strain–Life Relationships

**Figure 15:**
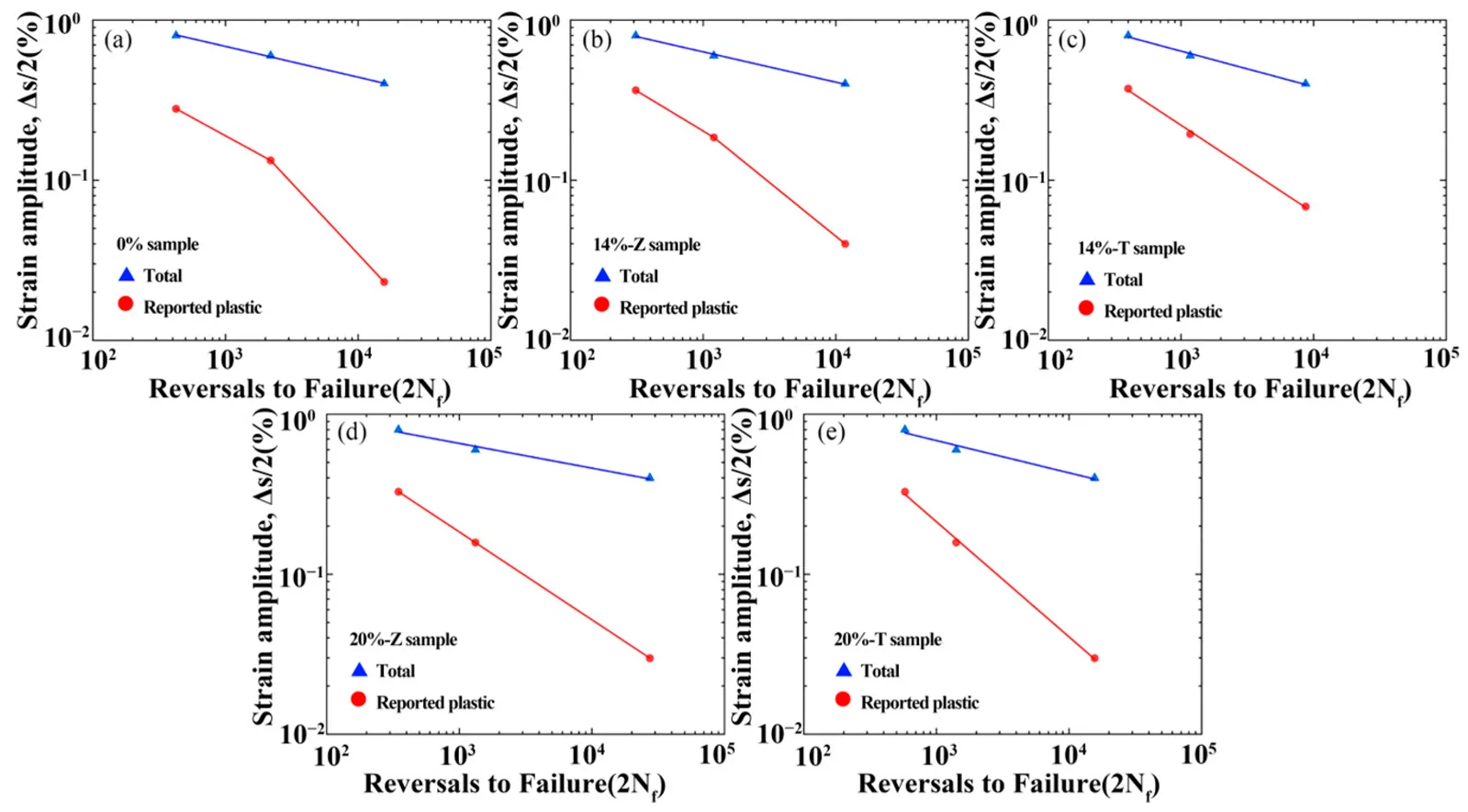
Total and half-life plastic strain–life relationships for (**a**) 0%; (**b**) 14%-Z; (**c**) 14%-T; (**d**) 20%-Z; and (**e**) 20%-T.

Figure 15 plots the total and reported half-life plastic strain amplitudes against 2N_f_, where N_f_ denotes the cycles to the recorded fatigue endpoint. Both amplitudes decreased with increasing reversals to the test endpoint. The connecting lines describe the observations within the tested range and are not regression fits.

Within the present three-amplitude dataset, 14%-T, 20%-Z, and 20%-T followed relatively smooth plastic strain–life trends, whereas the 0% and 14%-Z conditions showed more pronounced apparent changes in slope. These three-point trends describe strain-amplitude sensitivity. The direct-aged reference followed a substantially different processing route from the solution-treated, cold-drawn, and aged conditions and consequently may have a different sensitivity of plastic strain to strain amplitude. In addition, the LAGB fraction of 14%-Z was only 14.2%, far below the 49.9% of 20%-Z, consistent with differences in deformation substructure. Such differences may affect local slip, interfacial dislocation accumulation, and plastic-strain partitioning as the strain amplitude changes.

Taken together, the strain–life relationships, cyclic stress responses, half-life plastic strains, and dislocation structures indicate that the fatigue response to cold-drawing pre-deformation and short-time annealing depends on strain amplitude. At low strain amplitude, the long life of 20%-Z is consistent with a contribution from retained deformation substructures to dislocation storage and redistribution of localized plasticity. At high strain amplitude, stronger cyclic plasticity may increase the importance of strain accommodation, making partial substructure recovery a possible contributor to the longer life of 20%-T. The microstructure dependence of the strain–life response thus supports the proposed balance between constraint and accommodation.

These interpretations remain subject to fatigue and EBSD scatter and the three tested strain amplitudes. The differently processed reference and unequal post-fatigue histories limit causal comparisons. Matched 0.8% Z/T observations would help distinguish recovery-mediated accommodation from changes in precipitation or residual stress, while estimated exposure at 650 °C, including the pre-test hold, ranged from 0.844 to 15.699 h (Table 4), leaving oxidation-assisted damage as a competing contribution.

## 5. Conclusions

Using the direct-aged condition as a reference, this study established different initial microstructural states through 14% and 20% cold-drawing pre-deformation with or without short-time annealing at 930 °C for 10 min, and correlated these states with strain-controlled LCF behavior at 650 °C in air using EBSD, SEM, and TEM. The principal conclusions are as follows:

1. Cold-drawing pre-deformation altered the grain-boundary character and deformation substructure of GH4169. Increasing the pre-deformation from 14% to 20% was associated with an increase in the LAGB fraction from 14.2% to 49.9% in the Z condition and from 11.4% to 43.9% in the T condition. Short-time annealing at 930 °C for 10 min reduced the LAGB fraction of the 20% cold-drawn material from 49.9% to 43.9%, consistent with partial recovery of the deformation substructure, whereas the Σ3 twin-boundary fraction changed only slightly. These trends suggest that drawing level and subsequent annealing alter the retained deformation substructure, which may influence dislocation storage and local slip transfer.
2. All conditions exhibited predominantly cyclic softening at 650 °C, and the slowly softening regime generally shortened with increasing total strain amplitude. The recorded N_f_ ranking was strain-amplitude dependent: 20%-Z showed the highest recorded N_f_ at 0.4% (13,679 cycles), the direct-aged reference at 0.6% (1095 cycles), and 20%-T at 0.8% (289 cycles). The matched Z/T comparisons are consistent with a possible low-amplitude benefit of retained deformation substructures and a high-amplitude benefit of partial recovery for cyclic plastic accommodation.
3. The observed fatigue-damage features suggest strain-amplitude-dependent changes in localized cyclic plasticity. At 0.4%, 20%-Z was characterized by planar slip and dislocation pile-ups near grain boundaries and a particle tentatively assigned to δ. At 0.6%, planar slip remained visible, while more extensive tangles and vein-like structures were observed after a different fatigue history. Crack paths were associated with grain-orientation differences, boundary interactions, and heterogeneous local deformation. Together, the grain-boundary characteristics, cyclic hysteresis response, and selected dislocation structures support a proposed change from a low-amplitude contribution of retained deformation substructures to a possible high-amplitude benefit of partial recovery among the matched cold-drawn conditions.

## Figures and Tables

**Table 1:** Chemical composition of the GH4169 alloy bar (wt.%).

Cr	Fe	Nb	Mo	Ti	Al	Si	C	Mn	Co	Ni
18.97	18.17	5.30	3.02	1.02	0.49	0.07	0.031	0.02	0.01	Bal

**Table 2:** Processing routes applied to the GH4169 specimens.

Specimen	Processing Step			
	Solution Treatment	Diameter Reduction	Annealing	Aging
0%	—	—	—	720 °C × 8 h/FC to 620 °C + 620 °C × 8 h/AC
14%-Z	980 °C × 30 min/AC	14%	—	720 °C × 8 h/FC to 620 °C + 620 °C × 8 h/AC
14%-T	980 °C × 30 min/AC	14%	930 °C × 10 min/AC	720 °C × 8 h/FC to 620 °C + 620 °C × 8 h/AC
20%-Z	980 °C × 30 min/AC	20%	—	720 °C × 8 h/FC to 620 °C + 620 °C × 8 h/AC
20%-T	980 °C × 30 min/AC	20%	930 °C × 10 min/AC	720 °C × 8 h/FC to 620 °C + 620 °C × 8 h/AC

Note: AC denotes air cooling; FC denotes furnace cooling at 50 °C/h to 620 °C.

**Table 3:** Mean grain size and KAM of the investigated material conditions.

Condition	Mean Grain Size (μm)	Mean KAM (°)
0%	20.9	1.051
14%-Z	31.17	0.404
14%-T	30.71	0.428
20%-Z	35.14	0.729
20%-T	38.07	0.685

**Table 4:** Recorded fatigue life, N_f_ (cycles), and calculated cycling time (h, in brackets) at the indicated total strain amplitudes.

Condition	0.4%	0.6%	0.8%
0%	7858 [8.731]	1095 [1.825]	212 [0.471]
14%-Z	5884 [6.538]	601 [1.002]	155 [0.344]
14%-T	4360 [4.844]	587 [0.978]	200 [0.444]
20%-Z	13679 [15.199]	661 [1.102]	174 [0.387]
20%-T	7794 [8.660]	705 [1.175]	289 [0.642]

Note: Calculated cycling time is t = N_f_T/3600, with T = 4, 6, and 8 s at 0.4%, 0.6%, and 0.8%, respectively. The estimated duration at 650 °C includes a further 0.50 h pre-test hold and excludes heating, cooling, and post-fatigue tensile loading.

**Table 5:** Half-life plastic strain amplitudes (%).

Condition	At 0.4% Total	At 0.6% Total	At 0.8% Total
0%	0.0232	0.1336	0.2795
14%-Z	0.0399	0.1849	0.3643
14%-T	0.0684	0.1942	0.3734
20%-Z	0.0346	0.1585	0.3353
20%-T	0.0298	0.1580	0.3276

## Data Availability

The original contributions presented in this study are included in the article. Further inquiries can be directed to the corresponding authors.
